# Reactive oxidants and myeloperoxidase and their involvement in neutrophil extracellular traps

**DOI:** 10.3389/fimmu.2012.00424

**Published:** 2013-01-21

**Authors:** Heather Parker, Christine C. Winterbourn

**Affiliations:** Centre for Free Radical Research, Department of Pathology, University of Otago ChristchurchChristchurch, New Zealand

**Keywords:** superoxide, hydrogen peroxide, hypochlorous acid

## Abstract

Neutrophils release extracellular traps (NETs) in response to a variety of inflammatory stimuli. These structures are composed of a network of chromatin strands associated with a variety of neutrophil-derived proteins including the enzyme myeloperoxidase (MPO). Studies into the mechanisms leading to the formation of NETs indicate a complex process that differs according to the stimulus. With some stimuli an active nicotinamide adenine dinucleotide phosphate (NADPH) oxidase is required. However, assigning specific reactive oxygen species involved downstream of the oxidase is a difficult task and definitive proof for any single oxidant is still lacking. Pharmacological inhibition of MPO and the use of MPO-deficient neutrophils indicate active MPO is required with phorbol myristate acetate as a stimulus but not necessarily with bacteria. Reactive oxidants and MPO may also play a role in NET-mediated microbial killing. MPO is present on NETs and maintains activity at this site. Therefore, MPO has the potential to generate reactive oxidants in close proximity to trapped microorganisms and thus effect microbial killing. This brief review discusses current evidence for the involvement of reactive oxidants and MPO in NET formation and their potential contribution to NET antimicrobial activity.

## INTRODUCTION

Neutrophils release extracellular traps (NETs) in response to a diverse range of stimuli including a variety of microorganisms, microbial products, and chemokines (refer to the review by Guimaraes-Costa et al., 2012 for a more detailed list). NETs are composed of a scaffold of chromatin decorated with an assortment of neutrophil-derived proteins, including the enzyme myeloperoxidase (MPO; Urban et al., 2009). NETs are believed to contribute to host defense, supplementary to neutrophil phagocytosis, by trapping and potentially killing invading pathogens (Brinkmann et al., 2004). However, extended exposure of self-DNA and damaging neutrophil granule proteins may be detrimental to the host and NETs have been linked with autoimmunity (Kessenbrock et al., 2009; Lande et al., 2011) and other pathological conditions (Clark et al., 2007; Fuchs et al., 2010; Narasaraju et al., 2011; Caudrillier et al., 2012).

Activated neutrophils produce large amounts of superoxide (${\text{O}}_{2}^{•-}$) via their nicotinamide adenine dinucleotide phosphate (NADPH) oxidase. ${\text{O}}_{2}^{•-}$ dismutates to hydrogen peroxide (H_2_O_2_) leading to the formation of a variety of toxic oxygen derivatives, especially those formed by MPO-catalyzed reactions. Both the NADPH oxidase and MPO have been implicated in the regulation of NET formation. However, the specific reactive oxygen species (ROS) required remains to be clarified.

Myeloperoxidase catalyses the oxidation of chloride by H_2_O_2_ forming the strong oxidant hypochlorous acid (HOCl), the prime mediator of oxidative killing in the phagosome (Winterbourn and Kettle, 2012). MPO is present on NETs (Urban et al., 2009) and has the potential (given a supply of H_2_O_2_) to generate HOCl in close proximity to trapped bacteria, thus providing a prospective mechanism for oxidative NET-mediated killing. In this short review, we summarize experimental evidence for the involvement of ROS and MPO in the regulation of NET formation and discuss their potential contribution to NET antimicrobial activity.

## ROS AND MPO IN NET FORMATION

Studies into the mechanisms of NET formation (NETosis) indicate a complex process that differs depending on the stimulus. Given the variability in NET inducers (Guimaraes-Costa et al., 2012) the existence of more than one pathway is perhaps not surprising. The term NETosis is sometimes used to describe only those forms of NET formation associated with cell death (Steinberg and Grinstein, 2007), but NETs can be released from living cells (Yipp et al., 2012), and here we use NETosis to describe any form of NET formation. NETs differ with respect to composition, timing, the involvement of cell death and dependency on reactive oxidants (Clark et al., 2007; Fuchs et al., 2007; Yousefi et al., 2009; Pilsczek et al., 2010). To date, the majority of inducers examined show dependency on an active NADPH oxidase and there is evidence that with some stimuli MPO is also involved.

### NADPH OXIDASE DEPENDENCY

Evidence that an active NADPH oxidase is required for NET formation has come from studies using inhibitors of the oxidase, knockout mice, or neutrophils from patients with chronic granulomatous disease (CGD) whose NADPH oxidase is non-functional (Stasia and Li, 2008). Inhibition of the oxidase with diphenyleneiodonium chloride (DPI) prevents NETosis in response to several factors, including phorbol myristate acetate (PMA; Fuchs et al., 2007), an nitric oxide (NO) donor (Keshari et al., 2012), bacteria (Parker et al., 2012b), lipopolysaccharide (LPS; Yost et al., 2009), and complement factor 5a (C5a) after priming with granulocyte/macrophage colony-stimulating factor (GM-CSF; Yousefi et al., 2009). Interestingly with *Staphylococcus aureus*, an early phase of NET release induced by secreted bacterial products is independent of the oxidase and of cell death, with dependency on these increasing over time (Pilsczek et al., 2010). The later release of NETs was possibly induced by bacterial phagocytosis, which would have been slow under the conditions employed in this study. Thus, two different forms of NET stimulation could have operated over the course of the experiments. From this study it might be assumed that activation of the oxidase leads to NET expulsion by cell death and that the oxidase is not required for release from viable cells. However, oxidase-dependent NET release from living cells has been reported (Yousefi et al., 2009).

Strong evidence for NADPH oxidase-dependent NETosis comes from the finding that CGD neutrophils do not form NETs when stimulated with PMA, bacteria (Fuchs et al., 2007), or GM-CSF + C5a (Yousefi et al., 2009). Exogenously added H_2_O_2_ restores the ability of CGD neutrophils to produce NETs (Fuchs et al., 2007), as does gene therapy to reconstitute NADPH oxidase function (Bianchi et al., 2009). Using a mouse model of CGD, Ermert et al. (2009) found that *gp91*^-^^/^^-^ mice neutrophils do not make NETs when stimulated with PMA or *Candida albicans*. Furthermore, using genetically different inbred mouse strains these investigators observed that the level of NET formation correlated with the amount of ROS produced.

NET formation can also occur independently of oxidase activity. Not all stimulants activate the oxidase (Farley et al., 2012) and some that do may induce NETs independent of this. For example, the calcium ionophore ionomycin activates the NADPH oxidase yet induces NETs similarly in the presence or absence of DPI (Parker et al., 2012b). *S. aureus* leukocidins also induce NETs when oxidase activity is inhibited (Pilsczek et al., 2010). The oxidative burst was not measured in this study; however, similar concentrations of purified leukocidin combinations can induce ROS production (Colin and Monteil, 2003).

Although DPI is a general flavoenzyme inhibitor, the most likely explanation for its effect on NETosis is that it inhibits the NADPH oxidase, and this is supported by the CGD neutrophil and knockout mice studies. DPI does have other effects, including inhibition of mitochondrial complex I and inducible nitric oxide synthase (iNOS). However, even though an NO donor has been shown to induce NETs (Keshari et al., 2012), the low levels of iNOS in isolated human neutrophils make it unlikely that DPI prevents NETosis by inhibiting iNOS. Of note, a recent report describes DPI-sensitive NET induction by platelet activating factor, which does not activate the oxidase (Farley et al., 2012).

### THE ROLE OF MPO

**FIGURE 1:**
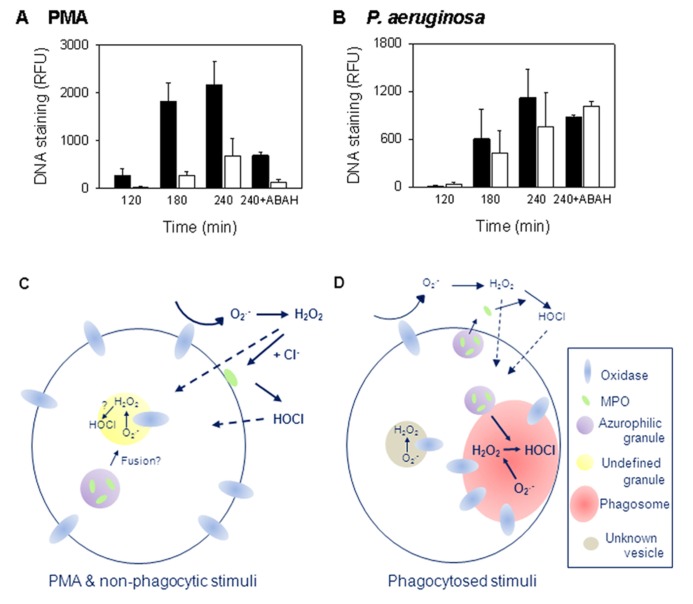
**Myeloperoxidase (MPO) is required for PMA but not bacterial induction of NETs**. **(A,B)** The release of NETs from control (filled bars) and MPO-deficient (open bars) neutrophils measured over 4 h. MPO-deficient neutrophils formed NETs less efficiently with PMA, but not with *P. aeruginosa*, than neutrophils from control donors. To inhibit MPO, samples were incubated in the presence of 100 μM of the MPO inhibitor 4-aminobenzoic acid hydrazide (ABAH). Results are means ± SEM of two to three independent experiments. For PMA, *p* = 0.02 at 180 min; *p* = 0.071 at 240 min by *t*-test. Data obtained with permission from Parker et al. (2012b). **(C,D)** Schematic representations of the intra- and extracellular locations of oxidant production in response to **(C)** soluble and non-phagocytic stimuli, or **(D)** phagocytosis (reviewed in Bylund et al., 2010 and Robinson, 2008). Details are given in the text. With PMA, oxidant production is predominately extracellular while phagocytosis induces largely intracellular production.

There is growing evidence that MPO is necessary for PMA-stimulated NETosis and the majority of studies indicate that an active enzyme is required. Inhibition of MPO decreases PMA-stimulated NETs (Akong-Moore et al., 2012; Palmer et al., 2012; Parker et al., 2012b) and neutrophils from MPO-deficient patients have reduced ability to produce NETs when stimulated with PMA. Metzler et al. (2011) found the level of NETs produced correlated with the degree of MPO deficiency and that neutrophils completely deficient in MPO could not make NETs. We observed just 3% of normal MPO activity was sufficient to allow PMA-induced NETosis (Parker et al., 2012b). Inhibition of this residual activity abrogated NET formation (**Figure 1A**).

Myeloperoxidase may not be required with all stimuli. We found inhibiting MPO in control donor neutrophils had no effect on *Pseudomonas aeruginosa*, *S. aureus*, or *Escherichia coli* NET induction (Parker et al., 2012b). MPO-deficient neutrophils also made NETs as efficiently as those from control donors when stimulated with *P. aeruginosa* and inhibition of residual MPO activity had no effect (**Figure 1B**; Parker et al., 2012b). In contrast to our observations, Akong-Moore et al. (2012) prevented *Pseudomonas*-induced NETosis with MPO inhibition. Our conditions favored phagocytosis (Parker et al., 2012b) and may account for the differences observed between the studies but this remains to be explored. Interestingly, MPO inhibition or knock out had no effect on NETosis in mouse neutrophils (Akong-Moore et al., 2012) indicating an apparent species-specific difference in NET formation. Of note, mouse neutrophils contain less MPO than human (Rausch and Moore, 1975).

Myeloperoxidase is reported to contribute toward NETosis, independent of its activity, by aiding chromatin decondensation (Papayannopoulos et al., 2010). Purified MPO increased nuclear decondensation in a cell-free system but the most dramatic increase occurred when MPO was added in conjunction with neutrophil elastase. In PMA-stimulated neutrophils, elastase translocated to the nucleus early in NETosis while MPO localized there later, when NET release was occurring (Papayannopoulos et al., 2010). Therefore, in neutrophils MPO may not play a direct role in chromatin decondensation.

To sum up, there is good evidence that MPO is important for PMA induction of NETs. From our studies, it would appear that this is not the case with bacteria. However, there are inconsistencies in the results from different laboratories that require explanation. Whether MPO is required with other physiological NET inducers is currently unknown. Nevertheless when MPO is needed, it appears that very little is actually required to facilitate NETosis.

### ASSIGNING THE SPECIFIC ROS REQUIRED

Activation of the neutrophil NADPH oxidase leads to the production of a variety of ROS. Assigning which are required for NETosis is not simple. The site of oxidase activation and degree of degranulation, which vary depending on the stimulus, affect the relative amounts of the different ROS produced as well as access to different cell constituents. With soluble stimuli, such as PMA, and non-phagocytosed particulate stimuli, activation largely occurs at the plasma membrane although some occurs at intracellular sites (reviewed in Bylund et al., 2010; **Figure 1C**). As yet these are not well characterized. During phagocytosis, activation mainly occurs at the phagosomal membrane (Winterbourn and Kettle, 2012), but electron microscope evidence shows that some also occurs elsewhere in the cell (Robinson, 2008; **Figure 1D**).

The NADPH oxidase removes electrons from cellular NADPH and transfers them across a membrane to oxygen, forming ${\text{O}}_{2}^{•-}$ in the extracellular environment, phagosome or a currently undefined intracellular compartment. ${\text{O}}_{2}^{•-}$ is membrane impermeable but rapidly dismutates to membrane permeable H_2_O_2_. Some of the H_2_O_2_ produced extracellularly may diffuse into the cell while some may react with MPO outside the cell (**Figures 1C**,**D**). The production of HOCl in the extracellular environment requires MPO release, the timing or level of which varies with stimulus. In the phagosome, due to high MPO concentrations, essentially all of the H_2_O_2_ should react with MPO before it can diffuse out (Winterbourn and Kettle, 2012). H_2_O_2_ can also react to form hydroxyl radicals and singlet oxygen (^1^O_2_). However, the generation of these oxidants by neutrophils is considered to be very low (Winterbourn and Kettle, 2012). PMA gives a larger, more sustained oxidative burst than other stimulants that induce NETs. However, even with PMA, oxidase activity is over well before NETs are released. ${\text{O}}_{2}^{•-}$ is produced within a minute of stimulation and continues for at least an hour but with the rate decreasing over this time (Decoursey and Ligeti, 2005). Similarly, oxidase activity continues for about 30 min following phagocytosis (Granfeldt and Dahlgren, 2001). Therefore, ROS produced must influence earlier rather than later events in NETosis.

By the nature of NADPH oxidase activation, it would seem it is likely that both the site of oxidant production and the nature of the oxidants produced are important in NET formation. Several groups have attempted to identify the specific ROS involved, primarily by using enzyme inhibitors or oxidant scavengers. One of the difficulties with this approach is targeting these to the appropriate compartment. It is straightforward to scavenge oxidants that are generated extracellularly. However, where there is intracellular oxidant production, as with PMA (Bylund et al., 2010), this is much more difficult to intercept. Consequently, there are still many uncertainties about what specific ROS generated by the NADPH oxidase or MPO are required in NETosis. The following sections discuss the evidence available for individual species.

#### Hydrogen peroxide

Several studies have shown that exogenously added H_2_O_2_ is sufficient to induce NETs (Fuchs et al., 2007; Neeli et al., 2009; Lim et al., 2011). However, addition of an oxidant and observation of NETs does not necessarily mean that this oxidant is responsible with physiological stimuli. With PMA, addition of catalase to scavenge extracellular H_2_O_2_ has little or no effect on NETosis (Fuchs et al., 2007; Parker et al., 2012b). It is plausible sufficient H_2_O_2_ is generated intracellularly to induce NETs so that extracellular scavenging would have minimal effect. This was examined using polyethylene glycol-catalase (PEG-catalase) which is taken up by endocytosis (Beckman et al., 1988), though its intracellular compartment is unknown. PEG-catalase reduced but did not completely inhibit PMA-NETosis while bacterial induction of NETs was unaffected (Parker et al., 2012b). Most likely PEG-catalase did not gain access to the appropriate intracellular sites to exert a full effect. Use of catalase inhibitors, such as azide or amino-triazole, has given inconsistent results (Fuchs et al., 2007; Palmer et al., 2012; Parker et al., 2012b). However, these also inhibit MPO, which complicates interpretation of effects.

#### Superoxide

Addition of superoxide dismutase (SOD) to neutrophils has been shown to modestly increase PMA-induced NETs (Palmer et al., 2012; Parker et al., 2012b). This would accelerate removal of extracellular ${\text{O}}_{2}^{•-}$ but have little effect on any generated intracellularly. Because most of the superoxide generated by neutrophils dismutates anyway, the presence of SOD would also make little difference to the amount of H_2_O_2_ produced (Winterbourn, 2008). At present we have no explanation for the SOD effect.

#### Hypochlorous acid and other MPO products

As the major strong oxidant produced by MPO, HOCl is a potential candidate for the oxidant responsible for MPO-dependent NET formation. Indeed, addition of HOCl to neutrophils has been reported to induce NETosis (Akong-Moore et al., 2012; Palmer et al., 2012). However, there are issues with interpreting these results. First, in our experience HOCl concentrations >50 μM are rapidly toxic to neutrophils (Carr and Winterbourn, 1997), whereas the concentrations used to induce NETs were several millimolar. Second, HOCl was added to RPMI which contains numerous scavengers, including >10 mM amino acids, which would consume the HOCl within seconds (Pattison and Davies, 2006). Although this would overcome toxicity, it would mean that very little HOCl would reach the neutrophils. Many products including amino acid chloramines would be formed, but it is unclear which would be responsible for NET formation. Third, addition of catalase to prevent extracellular HOCl formation, or removing HOCl with the potent scavenger methionine, did not inhibit PMA-stimulated NET formation (Parker et al., 2012b). Inhibition by >50 mM taurine was seen (Palmer et al., 2012), but interpretation of this observation depends on the specificity of these high concentrations. It is still possible that HOCl generated intracellularly could be involved, but more definitive evidence is needed before drawing this conclusion.

Alternative MPO products could be involved in NETosis. One example, singlet oxygen (^1^O_2_) has been implicated on the basis that NETs were observed after ^1^O_2_ was generated using irradiated Photofrin (Nishinaka et al., 2011). However, while it is theoretically possible for neutrophils to generate ^1^O_2_ from H_2_O_2_ and HOCl (Kiryu et al., 1999), it is a minor product (Hurst, 2012) and an unlikely candidate for NET regulation with other stimuli. MPO also catalyzes radical reactions, including lipid peroxidation. Interestingly, the radical scavenger Trolox inhibited PMA and LPS-induced NETosis in mouse neutrophils (Lim et al., 2011). This raises the possibility that a radical mechanism such as lipid peroxidation could be involved in the formation of NETs.

#### Summary of ROS required

In most cases, NADPH oxidase activity is needed for NET formation but the oxidants involved and their mechanisms of action are still unknown. The best, but not definitive, evidence is for H_2_O_2_ involvement, and with PMA a picture is emerging in which intracellularly generated MPO-derived ROS are important.

## INVOLVEMENT OF ROS AND MPO IN NET-MEDIATED MICROBIAL KILLING

It has been postulated that the role of NETs *in vivo* is to trap and kill microorganisms and there are some excellent scanning electron micrographs of NETs entrapping both bacteria and fungi (Brinkmann et al., 2004; Beiter et al., 2006; Bruns et al., 2010). The evidence for direct killing by NETs is less convincing (Nauseef, 2012). Most studies have examined NET killing by incubating pre-formed NETs with bacteria then diluting and plating. In some instances, failure to release bacteria from NETs may have been interpreted as killing, a problem we encountered but overcame with DNase treatment to degrade NETs (Parker et al., 2012a). Using this method, several groups (Bruns et al., 2010; Menegazzi et al., 2012; Parker et al., 2012a) have observed that NETs on their own do not kill *S. aureus*, *Aspergillus fumigatus* conidia, or *C. albicans* blastospores.

### EVIDENCE FOR MPO-MEDIATED NET KILLING

**FIGURE 2:**
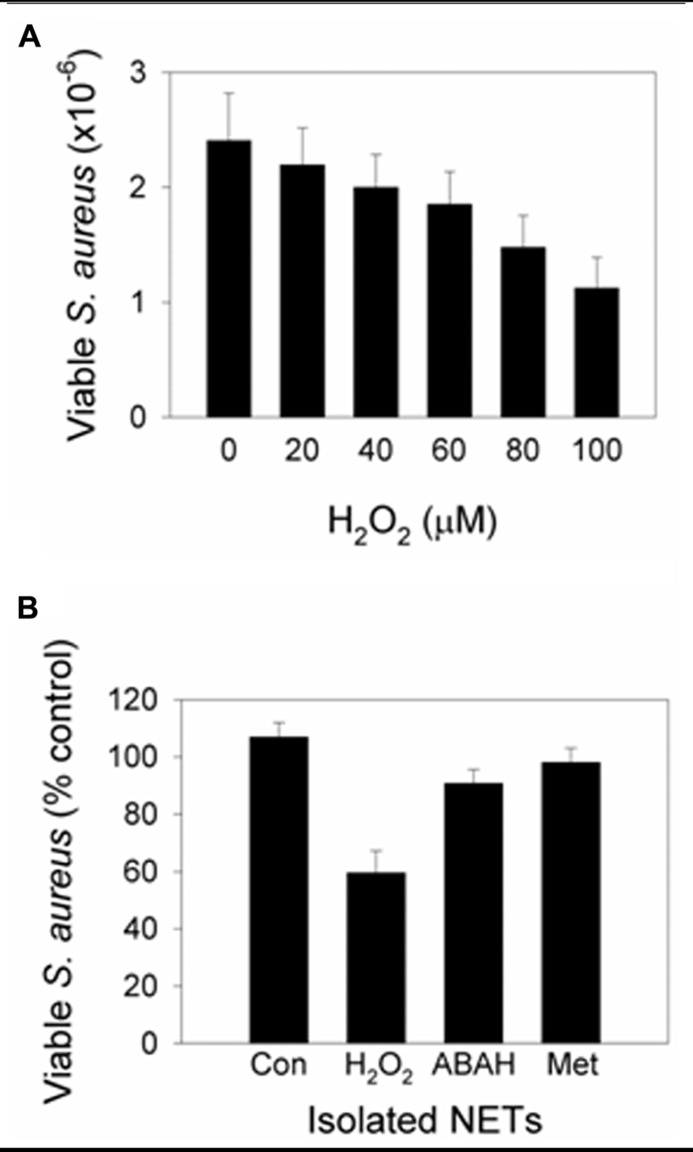
**Addition of H_2_O_2_ to NETs induces MPO-dependent killing**. Neutrophils were stimulated with PMA to form NETs then incubated with *S. aureus* in the presence or absence of **(A)** varying concentrations of H_2_O_2_ or **(B)** 100 μM H_2_O_2_ (added in 20 μM aliquots every 5 min to facilitate MPO turnover). At the examined concentrations, H_2_O_2_ in the absence of NETs had no significant effect on *S. aureus* viability. **(A)** Bacterial numbers significantly decreased with ≥40 μM H_2_O_2_ (*p*< 0.05, *t*-test on normalized data, *n* = 3). **(B)** Bacterial viability decreased with H_2_O_2_ (*p*< 0.001), and inhibition of MPO with ABAH and scavenging of HOCl with methionine (Met) prevented killing (*p*< 0.01; one-way ANOVA with Holm–Sidak pairwise comparison, *n* = 5). Results are presented as percent of control cells (Con) incubated with NETs alone. Data obtained with permission from Parker et al. (2012a).

Myeloperoxidase is present on NETs (Brinkmann et al., 2004; Urban et al., 2009; Parker et al., 2012a) placing it in close proximity to ensnared bacteria. NET-bound MPO is active and able to generate HOCl (Parker et al., 2012a). In our study, incubation of *S. aureus *with isolated NETs had no effect on bacterial viability. However, killing was observed when H_2_O_2_ was added as a substrate for MPO (**Figure 2A**). MPO inhibition and a potent HOCl scavenger prevented killing (**Figure 2B**). Therefore, NET-MPO has the potential to generate HOCl and effect microbial killing. At a site of inflammation, neutrophils that have formed NETs will no longer be producing ROS. However, during inflammation there is continued infiltration and activation of neutrophils which should provide the H_2_O_2_ required. The close proximity of NET-MPO to trapped microorganisms would be expected to facilitate exposure of microbes to lethal concentrations of HOCl and avoid all the oxidant being scavenged by the surrounding media. *In vivo* imaging using HOCl sensitive probes and differential fluorescent detection of live/dead bacteria would confirm if this occurs in living organisms.

## SUMMARY

There is good evidence that the enzymatic processes of the NADPH oxidase and MPO are important in NETosis but elucidation of the specific ROS and their reactions that regulate NET formation requires further investigation. While the use of scavengers and inhibitors is a useful aid to the study of ROS in NET formation, interpretation of results is confounded by limitations of specificity and getting sufficient concentrations to intracellular locales where the critical oxidant generation may occur. The intracellular pathways leading to chromatin decondensation and NET release are still being worked out. Once this information becomes available, the involvement of oxidants in individual steps can be investigated and a clearer picture should emerge.

## Conflict of Interest Statement

The authors declare that the research was conducted in the absence of any commercial or financial relationships that could be construed as a potential conflict of interest.
